# Increase the Surface PANI Occupancy of Electrospun PMMA/PANI Fibers: Effect of the Electrospinning Parameters on Surface Segregation

**DOI:** 10.3390/polym14163401

**Published:** 2022-08-19

**Authors:** Chen Qu, Peng Zhao, Yifan Ren, Chuandong Wu, Jiemin Liu

**Affiliations:** 1 School of Chemistry and Biological Engineering, University of Science and Technology Beijing, Beijing 100083, China; 2 Institute of Urban Safety and Environmental Science, Beijing Academy of Science and Technology, Beijing 100052, China

**Keywords:** electrospinning, process parameters, surface segregation, surface molecular occupancy

## Abstract

For preparing high-performance electrospun fibers with functional molecules that cannot cross-entangle themselves, such as conductive polymers, promoting the aggregation of functional molecules on the surface by surface segregation is a promising approach. In the present study, electrospun polymethyl methacrylate/polyaniline (PMMA/PANI) fibers were prepared under various conditions, including solution composition, applied voltage, tip-to-collector distance, temperature, humidity, and gas-phase solvent concentration, to examine the effects of the parameters on fiber morphology and surface segregation. The changes in fiber morphology and variations in the intensity of PANI and PMMA’s characteristic bands were investigated with scanning electron microscopy (SEM) and Raman spectroscopy. The results demonstrated that by changing the saturation difference and the viscosity, the amount of PMMA and PANI added significantly influenced whether surface segregation could occur. The effect of other investigated parameters on surface segregation was concluded to alter the molecular migratable time by affecting the jet flight time and the solvent volatilization rate. Among them, increasing the solvent concentration could significantly promote surface segregation without sacrificing morphological advantages. When the solvent concentration increased from 1.4 to 158 mg/m^3^, the Raman peak intensity ratio of PANI and PMMA increased from 2.91 to 5.05, while the fiber diameter remained essentially constant.

## 1. Introduction

Electrospinning is currently one of the most practical and feasible methods for the large-scale preparation of micro and nanofibers with uniform diameters and high specific surface areas [1]. Electrospun fibers can be made from virtually any soluble polymer, as well as biomolecules, metal oxide semiconductors, carbon compounds, and other nanomaterials [2,3,4,5,6]. Furthermore, diverse microstructures, such as nanowires, porous nanofibers, and nanotubes, can be manufactured to meet the needs of many domains, including air filtration, energy storage, and flexible electronic devices [7,8,9]. In solution electrospinning, the precursor solution flows out of the spinneret, the charges accumulate in the droplet, and then the droplet splits into fine jets under electrostatic repulsion and moves toward the grounded collector due to electric field forces during which the solvent evaporates and the jets shrink and solidify to form fibers [10,11]. To prevent the jets from breaking up into droplets in this process, the precursor solution must have a sufficiently high surface tension to resist the electrostatic stretching force [12,13].

For conductive polymers, biomolecules, and nanomaterials where molecular chain entanglement is not possible, electrospinning usually requires the addition of exogenous polymers to increase the surface tension of the solution [14,15]. The addition of the host polymer brings better spinnability to the solution, resulting in a good morphology and high specific surface area of the prepared fibers. The added polymers (host polymers) typically reach concentrations of 10 wt% to 15 wt% in the solution [15]. A large number of host polymers will encapsulate the functional molecules/materials inside the fibers, which seriously limits the overall performance of the fibers, especially for sensing materials, adsorbent materials, and other applications that rely on surface functional molecules/materials to provide active sites [16]. Surface segregation, an efficient method for surface modification of polymeric materials that enables surface enrichment of a specific component in a blended system, was anticipated to provide a solution to this dilemma [17,18]. In our previous study, by manipulating the saturation difference to promote surface segregation, electrospun polyacrylonitrile/polyaniline (PAN/PANI) fibers with abundant PANI molecules on the surface were successfully prepared, which greatly improved the gas-sensing performance of PAN/PANI fibers [17].

Surface segregation is a process influenced by multiple factors, including kinetics and thermodynamics working together at the same time [19,20]. Many previous reports have explored the mechanisms and factors influencing the occurrence of surface segregation, including solvent, ambient temperature, and polymers of the blended system, in addition to the previously mentioned saturation differences [18,21,22]. However, these reports concentrated on the surface segregation of bulk materials, whereas the solidification of the jet during electrospinning is a nearly transient process that differs significantly from that of bulk materials. Moreover, previous studies on the effects of electrospinning parameters have focused on their effects on fiber morphology and mechanical properties, with less concern for molecular distribution on the fiber surface. Thus, the factors affecting the surface segregation of electrospun jets are still unclear and need to be investigated for the preparation of high-performance electrospun fibers.

For electrospinning precursor solutions, the polymers employed depend on the target application of the fibers. The potential influencing factors to be explored are the concentration of different polymers, electrospinning process parameters, and environmental conditions. PANI has good electrical conductivity and has great potential in the field of sensors and flexible devices [23,24]. However, the poor processing performance due to its own rigid structure severely limits its application and performance [23]. The preparation of high-performance electrospun PANI fibers may greatly expand their applications. Polymethyl methacrylate (PMMA) has good solubility and mechanical properties and is often used in the preparation of high-performance electrospun fibers [25]. In the present study, electrospun polymethyl methacrylate/polyaniline (PMMA/PANI) fibers were prepared under various conditions, including solution composition, applied voltage, needle tip-to-collector distance, temperature, relative humidity, and gas-phase solvent concentration. Moreover, the morphology and fiber surface PANI occupancy of the fibers were analyzed to clarify the effect of various parameters on the fiber-forming and surface segregation processes.

## 2. Materials and Methods

### 2.1. Materials

Polymethyl methacrylate (average Mw ~120,000) was purchased from Sigma-Aldrich (St. Louis, MO, USA), polyaniline (emeraldine base, average Mw ~15,000, ≥97 wt%) and N, N-dimethylformamide (DMF, ≥99.9 wt%) were purchased from Macklin (Shanghai, China), and hydrochloric acid (HCl) was obtained from Sinopharm Chemical Reagent Co., Ltd (Shanghai, China). Before being used in the study, polyaniline was doped with a 1 mol/L HCl solution.

### 2.2. Electrospinning with Controlled Process Parameters

In this study, PMMA and PANI blended solutions were prepared for electrospinning to investigate the changes in fiber surface PANI occupancies. Due to the poor solubility of PANI, the demanded mass of PANI was preadded to DMF and sonicated for 1 h to promote its dispersion and dissolution. After that, PMMA was slowly added to the mixture while it was held at 40 °C with constant stirring for 12 h to assure homogeneity and spinnability of the final blended solutions. Except for PMMA addition, the containers were kept airtight to ensure a constant solution composition. The prepared solutions were then transferred into 5 mL plastic syringes tipped with a 23-gauge metallic blunt-edged needle and used for the preparation of electrospun PMMA/PANI fibers. The electrospinning was conducted with a commercial electrospinning apparatus (ET-1334H, Beijing Ucalery Technology Development Co., Ltd., Beijing, China). The syringe was mounted horizontally on the syringe pump and connected to a high-voltage power source. A grounded metal roller covered with aluminum foil was fixed horizontally in front of the syringe to collect the fibers at a speed of 140 rpm [17].

During electrospinning, the composition of the solution (mass fraction of PMMA, mass ratio of PANI to PMMA), process parameters (applied voltage, tip-to-collector distance TCD), and environmental conditions (temperature, humidity, and solvent concentration in the gas phase) were optimized and studied, as shown in Table 1. The study was conducted using the controlled variables method, where only the level of the parameter to be investigated was changed in each electrospinning group. The employed electrospinning apparatus had a closed chamber as well as temperature and humidity control modules, allowing for easy adjustment of all parameters except solvent vapor pressure. In routine use, the solvent vapor generated during electrospinning in the chamber could be expelled via the equipment’s air exchange module, maintaining a low concentration of solvent in the chamber. To investigate the effect of solvent concentration, the concentration of solvent in the chamber was artificially increased by bubbling DMF vapor into the chamber. The concentration of the solvent in the gas phase was determined by sampling-gas chromatography–mass spectrometry (GC–MS, Thermo Fisher ISQ1300, Waltham, MA, USA) analysis.

### 2.3. Morphological Characterization and Spectral Analysis

The prepared electrospun fibers were transferred to a vacuum oven and dried at 50 °C for 5 h to remove solvent residues before sampling and storage in a desiccator. The morphology of the electrospun fibers was characterized by scanning electron microscopy (SEM, ZEISS GeminiSEM 300, Oberkochen, Germany) to investigate the effect of the parameters on fiber diameter and surface morphology. The samples were coated with gold by the Oxford Quorum SC7620 sputter coater before SEM observation to obtain better observation results. The obtained SEM images were analyzed by ImageJ software (NIH) and the DiameterJ plugin to determine the average diameter [26]. To investigate the variation of molecular occupancy on the fiber surface, Raman spectroscopy was used to explore the characteristic peak intensity variation of the fiber samples, which was previously widely used for polymer surface analysis [27,28,29]. Raman characterization was conducted using a Raman spectrometer (HORIBA Scientific LabRAM HR Evolution, Kyoto, Japan) with an excitation wavelength of 633 nm. A PMMA-DMF solution with a mass fraction of 15 wt% was prepared and used to prepare electrospun PMMA fibers as standards for Raman spectroscopy analysis. Since PANI could not produce electrospun fibers independently, PANI powder was employed as the standard. All samples were replicated three times to minimize errors.

## 3. Results and Discussion

**Figure 1:**
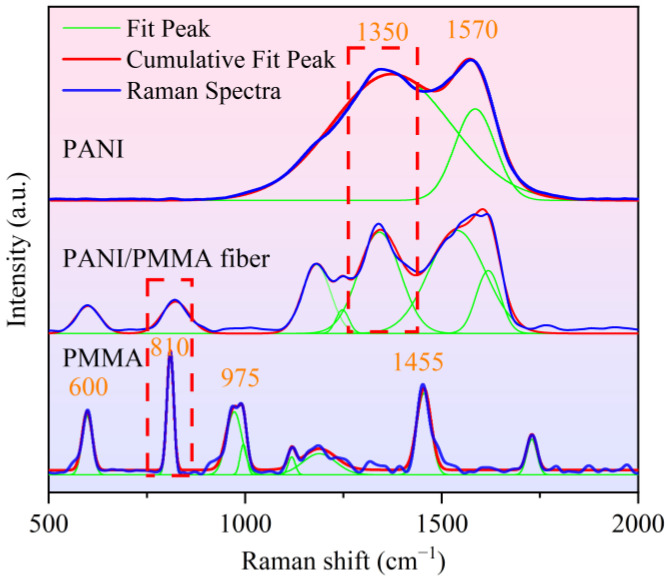
Raman spectra of PANI powders, PMMA fibers, and PMMA/PANI fibers.

Previous studies have revealed that increasing the contact probability between functional molecules and target molecules is the key to improving the performance of sensing materials [17,30]. Therefore, for fiber-based sensing materials, the morphology and the number of functional molecules on the fiber surface are crucial to the sensing performance. The morphological changes of the prepared fibers could be visually analyzed from SEM images, while precisely quantifying the PANI molecules on the fiber surface was complicated. In this study, the shifts in the characteristic peak intensity ratios of PANI and PMMA in Raman spectra of fiber samples were used to qualitatively evaluate the changes in the PANI occupancy on the fiber surface. The Raman spectra of the PANI powder, electrospun PMMA fiber, and PANI/PMMA fiber are displayed in Figure 1, along with the characteristic peaks labeled for PANI and PMMA. The characteristic peaks of doped PANI at 1350 and 1570 cm^−1^ could be attributed to C–N^+^ stretching of semiquinone radicals and C=C ring stretching of quinoid units [31]. PMMA’s characteristic peaks at 600, 810, 975, and 1455 cm^−1^ could be assigned to the C-C-O vibration, C-O-C vibration, C-H_3_ rocking, and C-H vibration of α-CH_3_, respectively [32]. Among these, the characteristic peaks corresponding to PANI and PMMA at wavelengths of 1350 cm^−1^ and 810 cm^−1^ in the Raman spectrum were chosen to calculate the intensity ratio, as they were weakly affected by other adjacent peaks or had higher peak intensity. The specific values of the ratios present in the following sections simply represent higher or lower PANI occupancy on the fiber surface and have no numerical association between them.

### 3.1. Effects of Solution Composition

Since the properties of the as-prepared material are closely related to the composition of the precursor solution, the effects of the mass fraction of PMMA in the precursor solution and the mass ratio of PMMA and PANI were first investigated and optimized.

#### 3.1.1. Mass Fraction of PMMA

**Figure 2:**
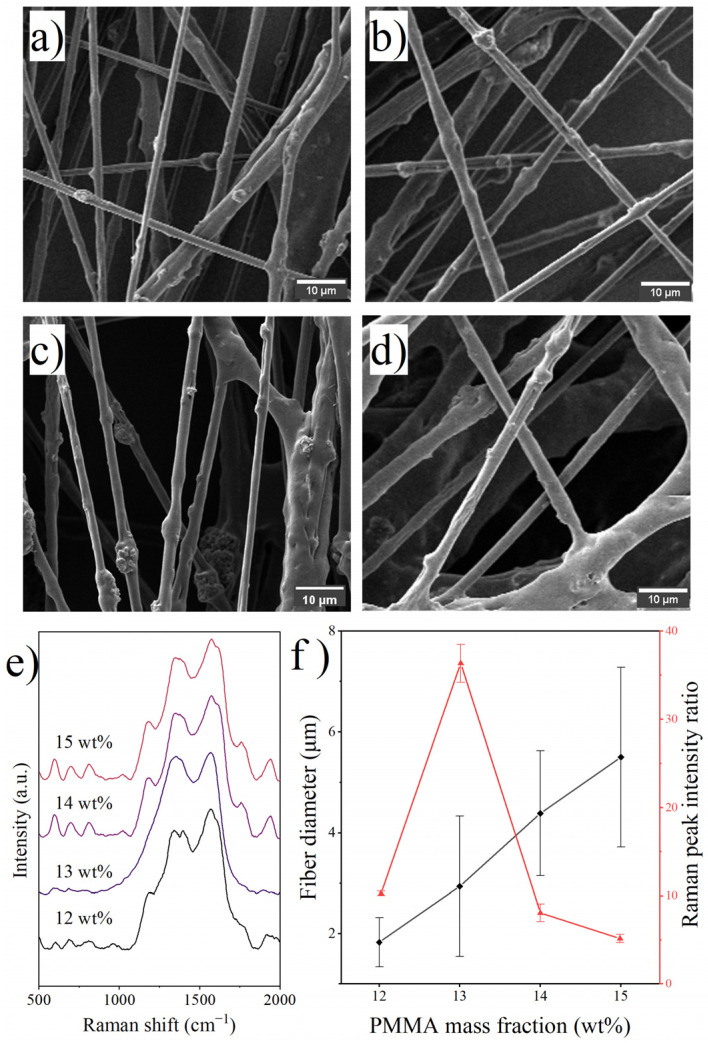
SEM images of fibers prepared with various PMMA mass fractions: (**a**) 12 wt%, (**b**) 13 wt%, (**c**) 14 wt%, (**d**) 15 wt%; (**e**) Raman spectra of the fibers and (**f**) effects of PMMA mass fraction on fiber diameter and surface PANI occupancy.

The mass fraction of PMMA in the solution ranged from 11 wt% to 15 wt%, with a constant mass ratio of PANI to PMMA of 0.4. The solution with a PMMA mass fraction of 11 wt% failed to be electrospun smoothly into fibers at 15 kV, presumably due to the low viscosity of the solution, which resulted in a jet surface tension less than the electrostatic stretching force [33,34]. As shown in Figure 2, the number of incompletely split jets and the average diameter of the generated fibers increased with increasing PMMA mass fraction due to the rapid rise in solution viscosity, leading to difficulty in jet splitting [34]. Previous studies have shown that components with low surface energy, small molecular weight, or linear molecular structure tend to appear more on the surface of the mixture in the mixture system [20]. That is, in the present study, PANI tended to aggregate on the surface. As evidenced by the Raman spectra, the PANI molecule occupancies on the fiber surface did not remain constant with the fixed PANI/PMMA ratio, suggesting that different levels of surface segregation occurred during the fiber forming process. When the PMMA mass fraction was 12 wt% and 13 wt%, surface segregation became more pronounced with more PANI molecules in the jet. However, when the PMMA mass fraction was too large, the entanglement of molecular chains became tight and complicated, considerably restricting the movement of PANI molecules and resulting in a decreased PANI occupancy on the fiber surface.

#### 3.1.2. PANI/PMMA Mass Ratio

**Figure 3:**
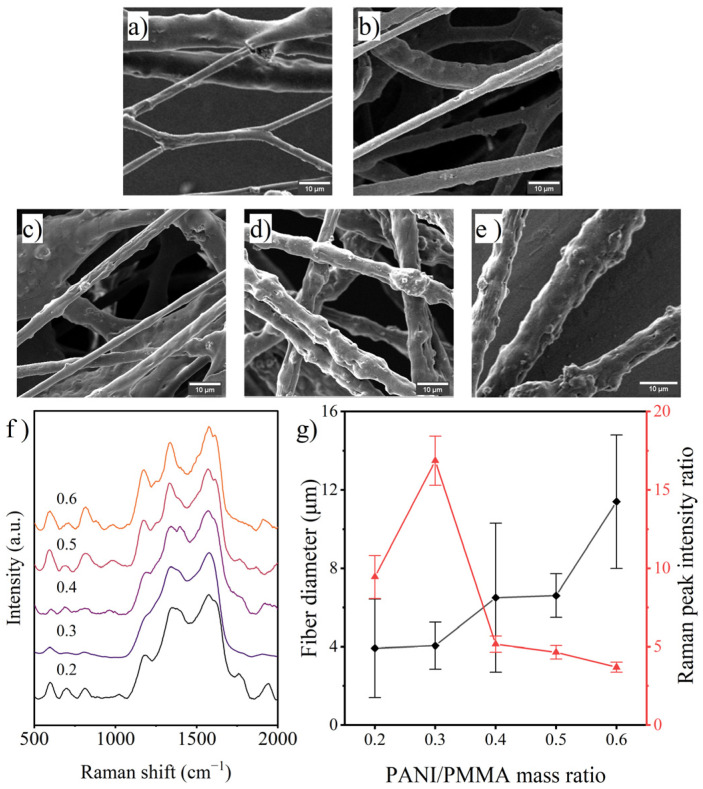
SEM images of fibers prepared with various PANI/PMMA mass ratios: (**a**) 0.2, (**b**) 0.3, (**c**) 0.4, (**d**) 0.5, (**e**) 0.6; (**f**) Raman spectra of the fibers and (**g**) effects of the PANI/PMMA mass ratio on fiber diameter and surface PANI occupancy.

In the investigation of the effect of the PANI/PMMA mass ratio, the mass fraction of PMMA in the solution was fixed at 15 wt%. Due to the high viscosity of the solution used, further increasing the amount of PANI added resulted in a rapid increase in the diameter of the fibers and a decrease in the uniformity of the diameter distribution, as shown in Figure 3. The PANI occupancy on the fiber surface showed a trend of increasing and then decreasing, indicating that increasing the amount of PANI added helped to increase the number of PANI molecules on the fiber surface, but when the addition of PANI caused a significant increase in solution viscosity, the decrease in molecular migration ability was more critical. To obtain the ideal material, the migratory ability of molecules within the solution must be ensured while increasing the saturation difference between the two components.

### 3.2. Effects of Process Parameters

In the investigation of the effects of process parameters, the optimized solution composition was used, i.e., PMMA mass fraction of 13 wt%, and PANI/PMMA mass ratio of 0.3.

#### 3.2.1. Applied Voltage

**Figure 4:**
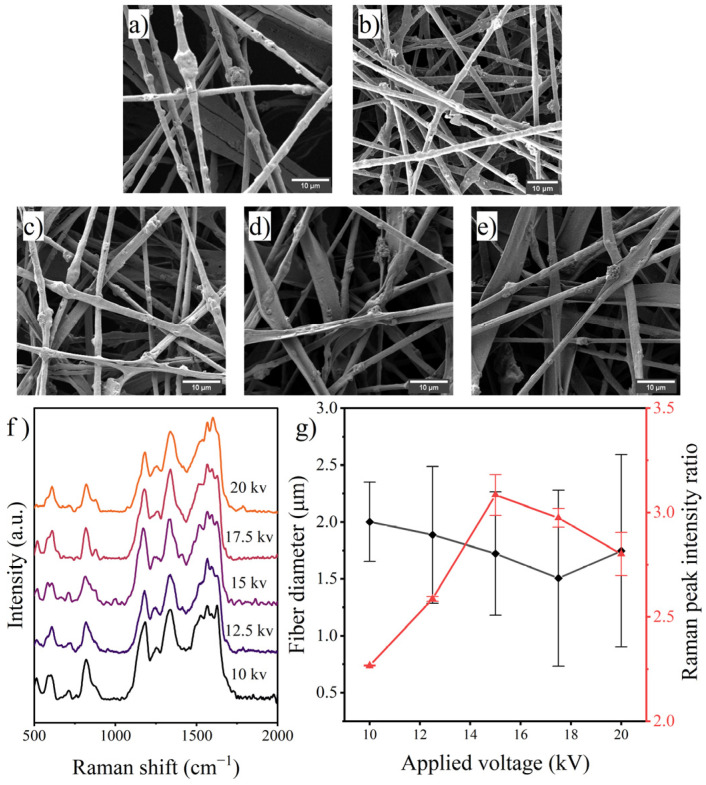
SEM images of fibers prepared with various applied voltages: (**a**) 10 kV, (**b**) 12.5 kV, (**c**) 15 kV, (**d**) 17.5 kV, (**e**) 20 kV; (**f**) Raman spectra of the fibers and (**g**) effects of applied voltage on fiber diameter and surface PANI occupancy.

Previous studies have demonstrated that an increase in voltage usually causes the jet to split into finer subjets, resulting in a decrease in fiber diameter. Meanwhile, the enhancement of the electric field shortens the flight time of the jet [35]. As shown in the SEM images in Figure 4, when connected to a lower voltage, part of the jet was not completely scattered, while with excessive voltage, the number of ribbon-like fibers increased. The average diameter of the fibers decreased slightly with increasing applied voltage, but when the voltage reached 20 kV, a slight increase in fiber diameter occurred, which could be attributed to an increase in the jet velocity and a reduction in the stretching time in the enhanced electric field; similar phenomena have been reported for cyclodextrin and poly(vinylidene fluoride) [36,37]. The Raman spectra of the prepared fibers showed that the PANI occupancy on the fiber surface peaked at 15 kV and then decreased with increasing voltage. In this regard, it is hypothesized that both decreasing the jet diameter and increasing the jet flight time will promote the aggregation of PANI molecules on the fiber surface.

#### 3.2.2. Tip to Collector Distance

**Figure 5:**
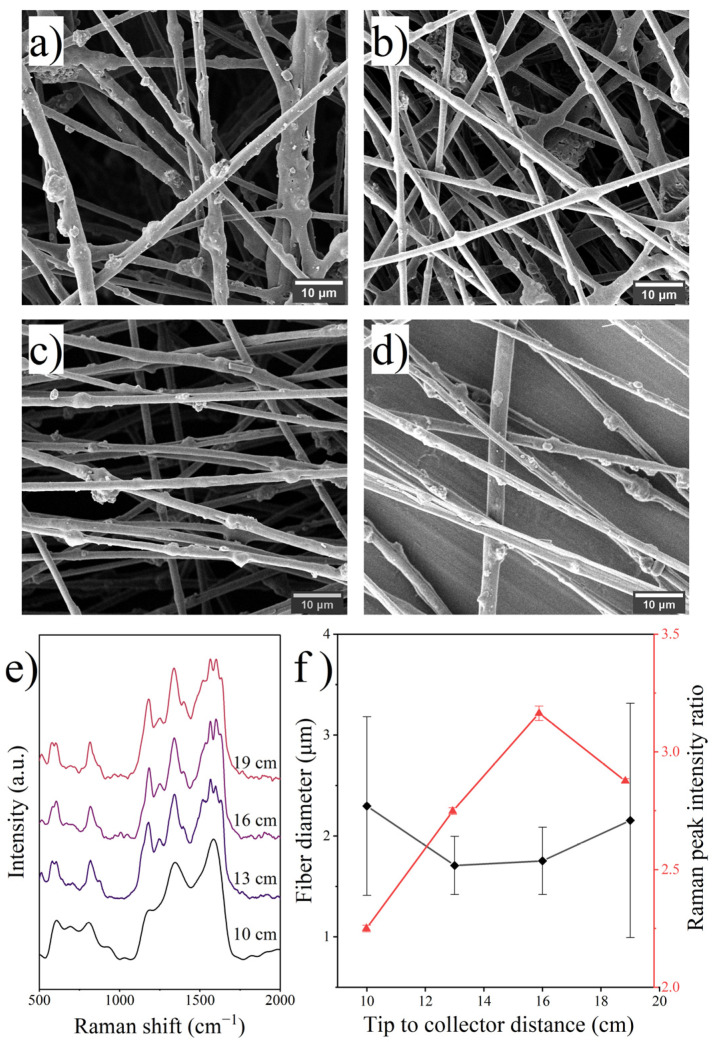
SEM images of fibers prepared with various TCD: (**a**) 10 cm, (**b**) 13 cm, (**c**) 16 cm, (**d**) 19 cm; (**e**) Raman spectra of the fibers and (**f**) effects of TCD on fiber diameter and surface PANI occupancy.

Changing the distance alters the electric field intensity in the electrospinning chamber, resulting in a change in the magnitude of the electrostatic stretching force on the jet and the jet flight time, which is closely related to solvent evaporation. As shown in Figure 5, the diameters of the fibers prepared at different distances were u-shaped and were close at 13 cm and 16 cm, and increasing or decreasing the distance would increase the fiber diameter. The increase in diameter of the fiber samples with TCDs of 10 cm and 19 cm was due to the shorter jet flight time and the weaker electric field, respectively. Raman analysis showed that, despite the close or larger fiber diameters, the PANI occupancies of the samples with TCD of 16 cm and 19 cm were higher than that of the sample with a TCD of 13 cm, indicating the significance of the extended jet flight time. However, the increase in jet diameter due to an oversized TCD would negatively affect the aggregation of PANI molecules on the fiber surface. The preparation of electrospun fibers with optimal performance may require a careful balance of these two influencing variables.

### 3.3. Effects of Ambient Parameters

#### 3.3.1. Working Temperature

**Figure 6:**
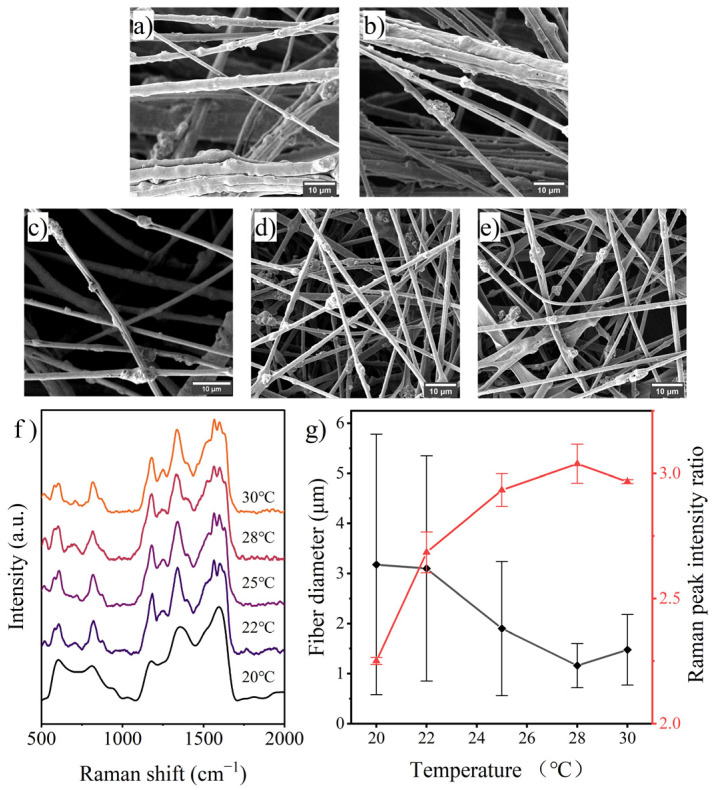
SEM images of fibers prepared with various working temperatures: (**a**) 20 °C, (**b**) 22 °C, (**c**) 25 °C, (**d**) 28 °C, (**e**) 30 °C; (**f**) Raman spectra of the fibers and (**g**) effects of working temperature on fiber diameter and surface PANI occupancy.

It is complicated how temperature affects electrospinning. On the one hand, increased temperature accelerates solvent evaporation and causes the jet to solidify more quickly, which is bad for shrinking fiber diameter and molecule migration in the jet. On the other hand, a temperature rise can efficiently lower the jet’s surface tension [38], which promotes molecular migration while also amplifying the jet’s stretching in the electric field and reducing the diameter of the fiber. According to the SEM images in Figure 6, for the same precursor solution, the decrease in working temperature raises the solution’s surface tension, causing the jet to fail to completely split and scatter, resulting in the formation of a multijet stacking morphology. The average diameter of the prepared fibers incrementally decreased between 20 and 28 °C, but the trend reversed at 30 °C, suggesting that the acceleration of solvent evaporation was more significant at higher temperatures. The PANI occupancy on the fiber surface increased throughout the 20 to 28 °C range, mirroring the fluctuation in fiber diameter. According to the results, it may be more advantageous to create desired materials with smaller diameters and more functional molecules on the fiber surface by electrospinning at a relatively high working temperature. A solvent with a high boiling point would be more appropriate in this scenario.

#### 3.3.2. Humidity

**Figure 7:**
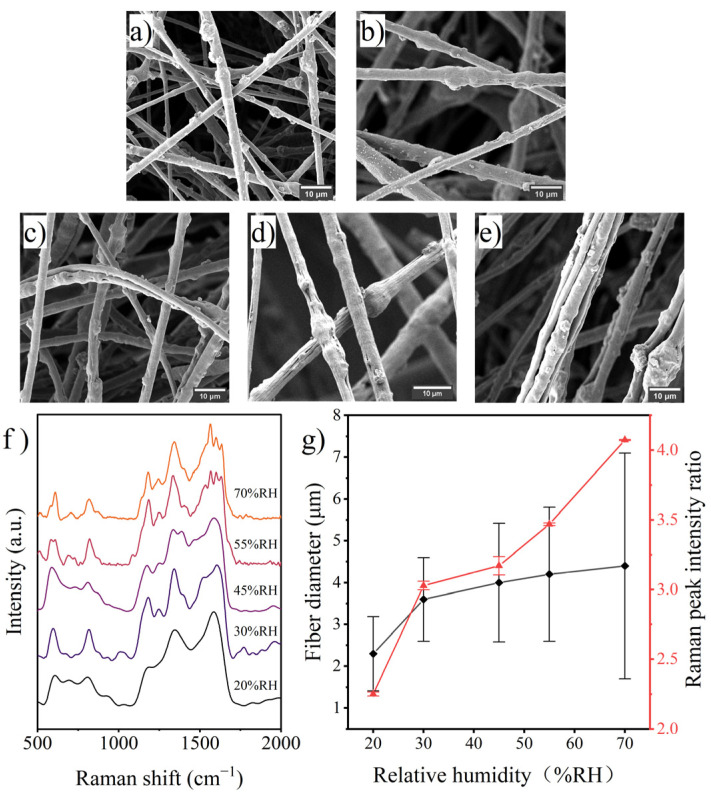
SEM images of fibers prepared with various humidity: (**a**) 20% RH, (**b**) 30% RH, (**c**) 45% RH, (**d**) 55% RH, (**e**) 70% RH; (**f**) Raman spectra of the fibers and (**g**) effects of humidity on fiber diameter and surface PANI occupancy.

Relative humidity has an important impact on the formation of electrospun fibers. First, water molecules near the Taylor cone will discharge the accumulated charge in the Taylor cone and change the splitting of the jet due to electric repulsion; second, water molecules affect the evaporation of the solvent and change the molecular crystalline state and surface morphology of the resulting fibers [39]; and finally, environmental humidity may affect the fiber collection stack [40]. In this study, the diameter of the prepared fibers increased sharply with increasing humidity, as shown in Figure 7. This result could be ascribed to the inhibitory effect of water molecules on jet spitting and solvent evaporation since the fiber thickened and the quantity of the multijet-fused fibers rose with humidity in the SEM images. Moreover, water molecules slowed down the solvent volatilization [41], which in turn prolonged the time for PANI molecule migration within the jet. As shown in Figure 6g, the surface PANI occupancy of the prepared fibers increased significantly with humidity.

#### 3.3.3. Solvent Concentration

**Figure 8:**
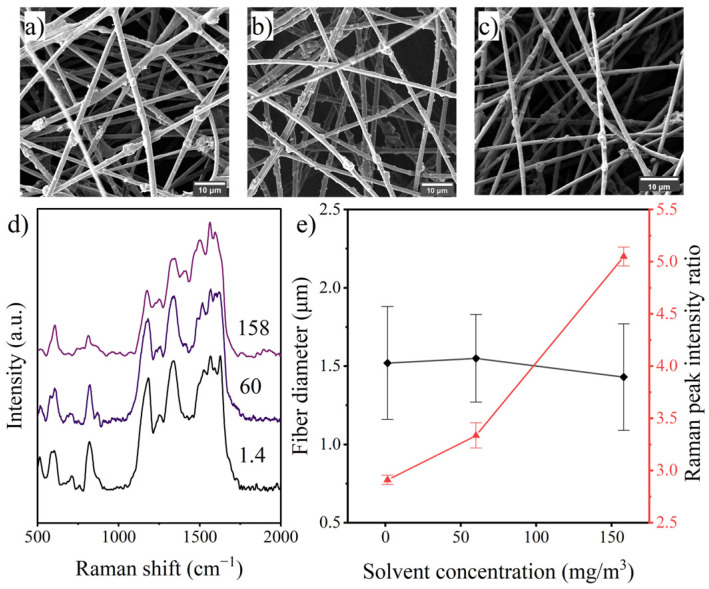
SEM images of fibers prepared with solvent concentrations of (**a**) 1.4 mg/m^3^, (**b**) 60 mg/m^3^, (**c**) 158 mg/m^3^; (**d**) Raman spectra of the fibers and (**e**) effects of solvent concentration on fiber diameter and surface PANI occupancy.

The previous results all indicate that there is a potential correlation between the surface PANI occupancy and the rate of solvent volatilization, and for this reason, the effect of solvent concentration in the gas phase on electrospinning was investigated. As shown in Figure 8, the PANI occupancy on the fiber surface of the prepared fibers significantly rose, while the fiber diameter decreased marginally with increasing solvent concentration in the environment. These results suggested that increasing the gas-phase solvent concentration could reduce jet solvent evaporation and slow jet solidification, thus enhancing jet stretching and prolonging the migration time of PANI within the jet. Naturally, if the solvent concentration is too high and exceeds a certain threshold value, it might lead to the appearance of jet-fused fibers or electrospinning failure.

### 3.4. Systematic Analysis

By investigating the effect of solution composition, the results showed that increasing the number of surface modificative molecules added (saturation difference) indeed promotes the occurrence of surface segregation, but at the same time, the confinement of molecules caused by the increase in solution viscosity will be increasingly significant, and this finding is consistent with Rafael’s report [20]. The effect of electrospinning parameters on the degree of PANI surface segregation was relatively modest compared to the environmental parameters. However, it was not the case that the occurrence of surface segregation was independent of the applied voltage as described in the previous study [18]. This is because electrospinning is a multifactor interrelated process where the applied voltage will change the jet coarseness and time of flight, which are related to the jet solidification speed and thus affect the degree of surface segregation. In addition, experimental results varying the applied voltage showed that finer jet diameters are favorable to improving surface segregation. The effect of TCD on surface segregation was also related to jet flight time. Among the environmental factors, the effect of temperature on surface segregation is concerned with altering the molecular migration ability and the rate of solvent volatilization, while humidity and gas-phase solvent concentration promote surface segregation by decreasing the solvent volatilization rate and extending the molecular migratable time. The paradoxical effect of increased temperature on surface segregation makes its effect on surface segregation relatively mild. The effects of relative humidity and gas-phase solvent concentration on surface segregation were the most pronounced of all the parameters investigated, but the negative effect of humidity on the morphology of the prepared fibers could not be neglected either. Overall, the saturation difference between the functional molecules and the host polymer is the driving force for the segregation, while the difficulty of molecular migration in the jet solution and the speed of jet solidification determine whether segregation can occur.

## 4. Conclusions

For composite electrospun fibers, fiber morphology and the number of functional molecules on the fiber surface are critical to the material’s overall performance. However, the parameters influencing electrospinning are complex and interrelated. This study used the control variable method to investigate the effects of solution composition, process parameters, and environmental parameters on the fiber morphology and surface PANI occupancy. The variations in fiber diameter and surface molecule occupancy caused by the investigated parameters were shown not to be monotonic, and optimum conditions existed. Among them, an appropriate solution composition was the basis for the preparation of ideal materials, and the gas-phase solvent concentration had the most significant effect on the surface molecule occupancy. Rational regulation of solution viscosity, saturation difference, jet splitting, and jet solidification speed by adjusting electrospinning parameters helps to prepare electrospun fibers with ideal morphology and abundant functional molecules on the surface.

## Figures and Tables

**Table 1:** Electrospinning parameter descriptions.

Investigated Parameters		Values
Solution composition	PMMA mass fraction (wt%)	11, 12, 13, 14, 15
	PANI/PMMA mass ratio	0.2, 0.3, 0.4, 0.5, 0.6
Process parameters	Applied voltage (kV)	10, 12.5, 15, 17.5, 20
	Tip-to-collector distance (cm)	10, 13, 15, 16, 19
Ambient parameters	Temperature (°C)	20, 22, 25, 30, 32
	Relative Humidity (%RH)	20, 30, 45, 60, 70
	Solvent concentration (mg/m^3^)	1.4, 60, 158

## Data Availability

Not applicable.
